# Veterinary Healthcare Provision and Quality of Reported Data on Antimicrobial Use in the Treatment of Livestock in Sierra Leone, 2016–2019

**DOI:** 10.3390/tropicalmed6020073

**Published:** 2021-05-10

**Authors:** Amara Leno, Walter Kizito, Amadu Tejan Jalloh, Mohamed Alpha Bah, Sorie Mohamed Kamara, Maria Zolfo, Amara Aidara Sheriff, Katrina Hann, Pruthu Thekkur, Ajay M. V. Kumar

**Affiliations:** 1 Livestock and Veterinary Services Division, Ministry of Agriculture and Forestry, Freetown, Sierra Leone; jallohtejan770@yahoo.com (A.T.J.); medalphabah2014@gmail.com (M.A.B.); 2 Emergency Center for Transboundary Animal Diseases, Food and Agriculture Organization of the United Nation, Freetown, Sierra Leone; 3 Médecins Sans Frontières, 1050 Brussels, Belgium; Walter.Kizito@brussels.msf.org; 4 Chief Agriculture Office, Ministry of Agriculture and Forestry, Freetown, Sierra Leone; soriesl@yahoo.com (S.M.K.); Hajamatifa@yahoo.com (A.A.S.); 5 Institute of Tropical Medicine, 2000 Antwerp, Belgium; mzolfo@itg.be; 6 Sustainable Health Systems, Freetown, Sierra Leone; hann.katrina@gmail.com; 7 International Union Against Tuberculosis and Lung Disease (The Union), 75006 Paris, France; Pruthu.TK@theunion.org (P.T.); akumar@theunion.org (A.M.V.K.); 8 The Union South-East Asia Office, New Delhi 110016, India; 9 Yenepoya Medical College, Yenepoya (Deemed to Be University), Mangaluru 575018, India

**Keywords:** One Health, antimicrobial resistance, data quality, operational research, SORT IT, low- and middle-income countries

## Abstract

Antimicrobials help in the prevention and treatment of infections and are crucial for animal production, but overuse can result in antimicrobial resistance. Hence, understanding data quality on livestock antimicrobial use is essential. We assessed frequency of reporting, completeness, and concordance of reported data and availability of human resources and infrastructure in 14 districts in Sierra Leone. This was a cross-sectional study involving a review of district and sub-district animal treatment forms submitted from January 2016 to August 2019. Out of the 14 districts, only 3 had filled forms available for review: A total of 6 (0.97% of 616 expected) district forms and 79 (1.15% of 6840 expected) sub-district forms. Data between district and sub-district treatment forms were fully discordant. Hence, completeness of data could not be assessed. All districts had livestock officers (barring one) and livestock assistants but no veterinarians. The gap in community animal health workers ranged from 14 to 100% per district. No districts had a functional computer or internet access. Reporting was non-existent in 11 districts and poor in the other 3. Resources are urgently needed to address critical gaps in human resources and capacity and computer and Internet connectivity to develop critical One Health surveillance functions at the national and sub-national levels.

## 1. Introduction

Antimicrobials help in the prevention and treatment of infections and play a critical role in animal production. Hence, their use is necessary for food security and animal welfare. However, overuse and misuse of antimicrobials has been associated with the spread of antimicrobial resistance (AMR), threatening both human and animal health, food security, and sustainable development [1]. In recent years, the impact of the use of antimicrobials in food-producing animals has been the subject of public debates, and the World Organization for Animal Health (Office International des Epizooties, OIE) has highlighted the need to regulate antimicrobial use in order to prevent the emergence and spread of resistant organisms in humans and animals and into the environment [2]. A systematic review and meta-analysis conducted in 2017 showed that interventions that restricted antibiotic use in food-producing animals were associated with a reduction in antibiotic-resistant bacteria in these animals and in turn reduced the potential for resistance in human populations, especially those who are in direct exposure to such food-producing animals [3].

Regular and detailed information about antimicrobial use in livestock is necessary for the evaluation of prescribing practices and for recommendations regarding appropriate use [4]. A survey conducted by the OIE in 2012 indicated that only 27% of the OIE member countries had an official system for collecting quantitative data on antimicrobial use in livestock [4]. The OIE recommendations following this survey were to (i) develop and set up an official harmonized national system for data collection to monitor AMR in relevant animal pathogens, and (ii) to detail the antimicrobials used in livestock, with the goal of creating a global monitoring database hosted by the OIE [4].

In high-resource settings, several programs for the monitoring of antimicrobial use and AMR in food-producing animals have been successfully implemented [4]. These include the European Surveillance of Veterinary Antimicrobial Consumption (ESVAC) implemented by the European Union [5]. However, the majority of low- and middle-income countries (LMICs) are still far behind, despite the existence of significant risk factors for communicable diseases and transmission of resistant pathogens. In Sierra Leone, like in many other LMICs, the use of antimicrobials in livestock treatment is unregulated and widespread [6].

The Sierra Leone government has recognized the magnitude of this problem and in 2017 conducted a situational analysis to provide baseline information on the nationwide AMR status, identifying gaps and challenges in curbing AMR [6]. This analysis found that the country had no national guidance on appropriate antimicrobial use and consumption in humans and animals. The country also has a severe shortage of veterinary officers (veterinary doctors or veterinarians) and veterinary drugs, including antibiotics [7]. Treatment of sick livestock in most parts of the country is carried out by district livestock officers, who are veterinary paraprofessionals with a basic training in veterinary medicine [6].

The Livestock and Veterinary Services Division of the Ministry of Agriculture and Forestry (MAF) has been collecting data on antimicrobial use in livestock since 2012, after conducting training on Epidemio-surveillance network for animal diseases (ENADIS) to facilitate the monitoring of antimicrobial use in the treatment of livestock [8]. As part of this reporting framework, the community animal health workers (who are veterinary paraprofessionals working at chiefdom level) are expected to collect data on a weekly basis from the chiefdoms (sub-district level units) and submit these to the district livestock officers, who are then expected to compile the reports on a monthly basis and submit them at the national level. Since the conception of this system, there has been no formal evaluation of the process and of the quality of data collected and available at MAF offices.

Understanding the quality of reported data on antimicrobial use in animals in Sierra Leone is an essential first step towards strengthening One Health surveillance and understanding the subsequent implications for potential AMR. The results from this study will be used to inform the MAF and the wider veterinary community about any identified gaps in the current data collection and reporting system, which can be used to strengthen the monitoring system.

Hence, we aimed to assess the frequency of reporting, completeness and concordance of reported data and availability of resources for reporting from the 14 districts of Sierra Leone, between January 2016 and August 2019. The specific objectives were to (i) assess reporting compliance from the district livestock offices; (ii) compare the concordance and completeness of reported data from the districts with data captured in the community animal health worker forms; and (iii) describe the current availability of required human resources and computer and Internet connectivity at reporting sites.

## 2. Materials and Methods

### 2.1. Study Design

This was a cross-sectional study.

### 2.2. General Setting

Sierra Leone is a West African country in the Atlantic Ocean with an estimated population of 7 million people [6]. The country borders the republic of Guinea from the east to the northwest and the republic of Liberia to the south. The country is divided into 16 districts (only 14 districts were present at the time of the study). Each district is further subdivided into chiefdoms, and each chiefdom consists of many villages. Agriculture (crops, livestock, forestry, and fisheries) is the mainstay of the country’s economy. It contributes to over 75% of livelihoods and about 47% of the country’s gross domestic product (GDP). Livestock agriculture contributes to about 6% of the total GDP [6]. The main livestock species for local meat production are cattle, goats, sheep, poultry, and pigs. More than 90% of these animals are indigenous. According to a survey conducted by the Food and Agriculture Organization of the United Nations in 2016, livestock estimates were 245,736 cattle, 963,001 sheep, 1,567,789 goats, 125,064 pigs and 14,721,718 poultry [6]. The livestock production system for ruminants has been described generally as traditional, free range, or extensive low input.

### 2.3. Specific Setting

The Livestock and Veterinary Services Division is one of the six divisions under the MAF with the mandate to promote animal health and production services. The division is formed by two units: (i) the Animal Health unit, in charge of animal health, animal disease surveillance including AMR, and (ii) the Animal Production unit, in charge of animal husbandry, animal traction (defined as use of animals to pull farm equipment, vehicles, and other loads) and animal production activities.

District livestock officers (one per district) are responsible for the treatment of sick animals in the country. They are assisted by livestock assistants (one per district) and a network of community animal health workers (one per chiefdom). Every district is expected to have two veterinarians as per the guidelines.

The data about antimicrobials used in the treatment of livestock are expected to be captured by the community animal health workers and livestock assistants in a prescribed format (hereafter referred to as the “sub-district treatment form”) and submitted to the respective district livestock officer on a weekly basis. The district livestock officers are expected to validate and compile the data on a monthly basis (hereafter referred to as the “district treatment form”) and submit them to the head of the epidemiology unit at the national level by the 28th of every month. Copies of the submitted reports are kept in the files of district livestock offices, and district treatment forms are maintained in files at the MAF national office. The variables in the sub-district treatment form include name, address and contact number of the animal owner, date of visit, animal and its sex, clinical diagnosis, treatment (includes details of antimicrobials, anti-parasitic drug, multivitamins, or any other treatment provided), payment, and remarks. The variables in the district form consist of the same variables included in the sub-district form, except the sex of the animal and details of payment. The formats of the district and sub-district treatment forms are provided in Appendix A.

### 2.4. Study Population 

This included all district treatment and sub-district treatment forms available at district livestock offices for the period of 1 January 2016–31 August 2019.

### 2.5. Data Variables, Sources of Data, and Data Collection 

Data collection was conducted by the principal investigator and his team members trained by him. Three data collection teams visited the national MAF and district livestock offices and collected copies of all the district treatment forms and sub-district treatment forms available for the study period. Data on the current availability of human resources (number recommended and number available for each cadre of staff) and computer (functional or not) and Internet connectivity (present or not) were also collected by consulting the district livestock officers. Data were collected from February to April 2020.

### 2.6. Data Analysis

Data analysis was performed using simple frequencies and percentages. To assess reporting compliance, we assessed whether the number of forms available during the study period matched against the expected number of forms. This was conducted separately for district treatment forms and sub-district treatment forms available at the district offices, as no forms were available at the national MAF office. Therefore, the data presented are the duplicates of district treatment forms found at the district level. To assess the concordance and completeness of data, we reviewed all of the sub-district treatment forms that corresponded to the same month of the district treatment form. If the total number of animals treated in the district treatment form for a given month matched with the sum of all the animals treated in each of the sub-district treatment forms for the corresponding month, then the data were considered complete. We also performed a qualitative document analysis of district treatment forms and identified missing variables and data inconsistencies. If all of the details (with regard to the animals treated, clinical diagnosis, and the treatment provided) in the district treatment form matched with the data in sub-district treatment forms, then the data were considered concordant. Otherwise, they were considered discordant.

## 3. Results

The frequency of availability of district treatment forms at district livestock offices is depicted in Table 1. Of the 14 districts, 11 (79%) districts did not have any forms available from the study period. The three districts that had forms available were Kenema (one form, 2017), Kambia (two forms, 2019), and Bombali (three forms, 2017), which is substantially lower than the total number of forms expected (n = 44) from each of the districts during the study period. Thus, of 616 forms expected in total during the study period, only 6 (0.97%) were available.

We also reviewed the sub-district treatment forms used by the community animal health workers to capture data from their respective chiefdoms, and the results are shown in Table 2. These forms were not present in 11 out of 14 (79%) districts. Only three districts (Kenema, Kambia, and Bombali) had these forms available, the number of which was far below that expected. Thus, of 6840 sub-district forms expected in total during the study period, only 79 (1.15%) were available. Comparing the district and sub-district treatment forms, we found that the data in the two forms were all discordant and, because of this reason, we could not assess the completeness of the data reported.

A qualitative document analysis of district treatment forms showed that the name of the reporting officer and the phone number of the animal owner were consistently absent in all of the forms. All the other fields, such as the name of the animal, clinical diagnosis, and treatment, were filled consistently. As per the information available in the six district treatment forms, a total of 77 animals were treated. Some of the most common antibiotics used were tetracycline (used 5 animals), oxytetracycline (used in 13 animals), tylosin (used in 7 animals), and sulfadimidine (used in 3 animals). The other common medicines provided were ivermectin (used in 29 animals), multivitamin tablets (used in 35 animals), oral rehydration salts (used in 3 animals), and oxytocin injection (used in 6 animals). The dose of the drugs given was not consistently mentioned. The duration of the treatment was not mentioned in any of the forms. The “remarks” column was either not filled or filled variably; while some forms mentioned “recovered” indicating the outcome of treatment, others simply mentioned “treated”.

The analysis of availability of human resources is shown in Table 3. All of the districts except Western Area Rural district had a district livestock officer in place. All of the districts had a livestock assistant as recommended. None of the districts had veterinarians. Overall, there were 72 community animal health workers in the country against 168 recommended (gap of 57%). This gap ranged from as low as 14% (Kailahun district) to as high as 100% (Western Area Rural district). All of the districts had a computer, but none of them were functional. None of the districts had Internet connectivity.

## 4. Discussion

Data collection and reporting on antimicrobial use are continuing challenges faced by the livestock and veterinary services divisions in Sierra Leone.

Our findings indicate that no data were available at the national level on livestock antibiotic uses, and, moreover, the scarce data available at the district level were not a true reflection of the data coming from the community. In addition, the availability and the completeness of the reported data were poor for almost all of the districts.

The analysis of available human resources in the livestock and veterinary services showed that (i) all but one district had a district livestock officer in place, and (ii) all of the districts had a livestock assistant as recommended, but (iii) none of the districts had veterinarians, pointing at the scarcity of available key human resources. In terms of infrastructure, while all districts had a computer, none of them were functional nor had Internet connectivity.

Available antimicrobial use data on the treatment of livestock show that current plans and actions are often based on limited information [9]. Antimicrobial consumption in animal production contexts in LMICs remains largely undocumented, limiting the ability to establish and monitor progress toward achieving consumption targets [10]. In responses to AMR, a national surveillance system is critical for monitoring total consumption for which effective policies can be introduced to curb excessive consumption [11]. Automating the transfer of data from one system to another would improve data incompleteness, eliminate transcription errors and delays in reporting, and reduce the reporting burden on human resources [12].

Combating AMR requires in-country multi-sectoral actions and global collective efforts using a “One Health” approach. The holistic approach of One Health requires not only interdisciplinary cooperation but standardized methods for communicating and archiving data, enabling participants to easily share what they have learned and allow others to build upon their findings [13].

This study is new and unique, because the availability and the quality of the reported data on the antimicrobial use in the treatment of livestock in Sierra Leone had not been previously assessed. However, the scarcity of data and the limited available cadres reporting on the data validity are considerable limitations for our findings. This study was carried out with difficulty and encountering many logistic challenges, such as COVID-19 domestic traveling restrictions, limited district accessibility, unavailability of livestock officers at the district sites, and erratic storage of the paper-based forms. Another limitation of our study is that we focused on the antimicrobial use and reporting in the government sector but not in the private sector. The private sector accounts for ~85% of all antimicrobials procured and used in Sierra Leone, and this procurement process is not regulated by the government. Future studies should make efforts to collect data on sales at drug stores, which may give a better picture of the overall consumption of antimicrobials in the country.

The collection of the data by the livestock officers was also challenging at the district level, because some forms were still at the community level, in the hands of the community animal health workers, or behind schedule with their weekly report and submission. No automated data collection or transfer was available at the district level, hampering the correct collection and report of the monthly data at the national level.

This paper identifies major gaps in human resources and the infrastructure for the production, collection, and use of data essential for One Health AMR surveillance and the cornerstone in the implementation of a national action plan on AMR. The study is an eye opener to the need to make resources available for the strengthening of monitoring and for evaluation system development at the MAF. It also opens the door to further studies on barriers and enablers for the issues related to data recording and transmission identified in our results. A skilled monitoring and evaluation officer at the national level could develop a comprehensive and unique reporting form, from the district to the national level, as well as designing the requisite system for data collection and use. The availability of dedicated cadres, with adjunct training and supervision, at the district and national level for data collection and the availability of infrastructures are of considerable importance.

## 5. Conclusions

To the best of our knowledge, this is the first study on the quality of reported data on antimicrobial use in the treatment of livestock in Sierra Leone. Given the findings of the review, it seems clear that no data were available at the national level on livestock antibiotic uses, and, moreover, the scarce data available at the district level were not a true reflection of the data coming from the community. The frequency of reporting and the completeness of the reported data were poor for almost all of the districts and pose a serious threat to food safety and security on this continent. We therefore strongly recommend that resources be directed to support the strengthening of surveillance systems at the MAF in order to support the One Health approach and recommendations advocated by the WHO, OIE, and FAO.

## Figures and Tables

**Table 1:** Number of monthly district treatment forms expected and available by district in Sierra Leone from January 2016 to August 2019.

Region	District	Expected Number of Forms for the Study Period	Number of Forms Available			
			2016	2017	2018	2019
East	Kailahun	44	0	0	0	0
	Kono	44	0	0	0	0
	Kenema	44	0	1	0	0
West	Western area urban	44	0	0	0	0
	Western area rural	44	0	0	0	0
North	Bombali	44	0	3	0	0
	Kambia	44	0	0	0	2
	Koinadugu	44	0	0	0	0
	Port Loko	44	0	0	0	0
	Tonkolili	44	0	0	0	0
South	Bo	44	0	0	0	0
	Bonthe	44	0	0	0	0
	Moyamba	44	0	0	0	0
	Pujehun	44	0	0	0	0
Total		616	0	4	0	2

**Table 2:** Number of weekly sub-district treatment forms expected and available per district in three districts of Sierra Leone from January 2016 to August 2019.

District	Number of Chiefdoms	Expected Forms per Year (One Form per Chiefdom per Week)#	Number of Sub-District Treatment Forms Available			
			2016, n (%) *	2017, n (%) *	2018, n (%) *	2019, n (%) *
Kenema	16	832	4 (0.5)	4 (0.5)	5 (0.6)	8 (1.6)
Kambia	7	364	6 (1.6)	10 (2.7)	7 (1.9)	3 (1.3)
Bombali	13	676	11 (1.6)	10 (1.5)	10 (1.5)	1 (0.2)

* Percentage = number of forms received each year/expected number of forms per year; expected = number of chiefdoms × 52 (weeks per year). For the year 2019 (January to August), expected = number of chiefdoms × 32 weeks.

**Table 3:** Human resources at the district livestock offices of Livestock and Veterinary Services Division of the Ministry of Agriculture and Forestry, Sierra Leone, as of February 2020.

District	District Livestock Officers		Livestock Assistant		Veterinarians		Community Animal Health Workers		
	Rec	Aval	Rec	Aval	Rec	Aval	Rec	Aval	% Gap
Bo	1	1	1	1	2	0	14	5	64
Bombali	1	1	1	1	2	0	13	4	69
Bonthe	1	1	1	1	2	0	11	9	18
Kailahun	1	1	1	1	2	0	14	12	14
Kenema	1	1	1	1	2	0	16	4	75
Koinadugu	1	1	1	1	2	0	11	6	45
Kambia	1	1	1	1	2	0	7	3	57
Kono	1	1	1	1	2	0	14	8	43
Moyamba	1	1	1	1	2	0	14	1	93
Port Loko	1	1	1	1	2	0	11	5	55
Pujehun	1	1	1	1	2	0	12	3	75
Tonkolili	1	1	1	1	2	0	11	9	18
Western area urban	1	1	1	1	2	0	10	3	85
Western area rural	1	0	1	1	2	0	10	0	100
Total	14	13	14	14	28	0	168	72	57

Rec = recommended, Aval = available, % gap = gap percentage ([recommended − available × 100]/recommended).

**Figure A1:**
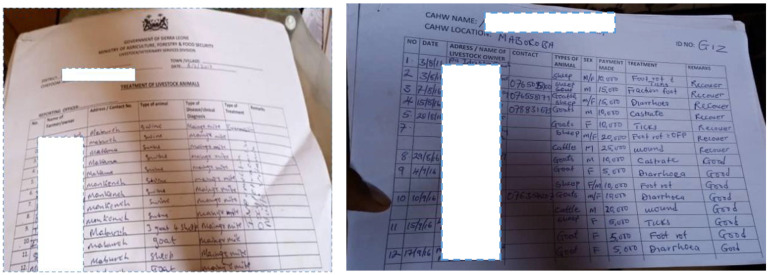
Formats of the district and sub-district treatment forms.

## Data Availability

All of the data that concern the content of this paper are present in the paper.

## Open Access Statement and Disclaimer

In accordance with WHO’s open-access publication policy for all work funded by WHO or authored/co-authored by WHO staff members, WHO retains the copyright of this publication through a Creative Commons Attribution IGO license (http://creativecommons.org/licenses/by/3.0/igo/legalcode, accessed on 7 May 2021), which permits unrestricted use, distribution, and reproduction in any medium provided the original work is properly cited. There should be no suggestion that WHO endorses any specific organization, products, or services. The views expressed in this article are those of the authors and do not necessarily reflect those of their affiliated institutions. The use of the WHO logo is not permitted. This notice should be preserved along with the article’s original URL.
